# Associations of physical activity levels, and attitudes towards physical activity with blood pressure among adults with high blood pressure in Bangladesh

**DOI:** 10.1371/journal.pone.0280879

**Published:** 2023-02-03

**Authors:** Fakir M. Amirul Islam, Mohammad Ariful Islam, Mohammad Arzan Hosen, Elisabeth A. Lambert, Ralph Maddison, Gavin W. Lambert, Bruce R. Thompson

**Affiliations:** 1 School of Health Sciences, Swinburne University of Technology, Hawthorn, VIC, Australia; 2 Organisation for Rural Community Development (ORCD), Dariapur, Narail, Bangladesh; 3 Iverson Health Innovation Research Institute, Swinburne University of Technology, Hawthorn, VIC, Australia; 4 Faculty of Health, Institute for Physical Activity and Nutrition (IPAN), Deakin University, Geelong, VIC, Australia; University of Liverpool, UNITED KINGDOM

## Abstract

**Background:**

Physical activity is important for the control of high blood pressure (hypertension). We aimed to investigate the associations of current physical activity levels, sedentary time, knowledge of and attitude towards physical activity with blood pressure in people with hypertension in a rural area in Bangladesh.

**Methods:**

A total of 307 adults aged 30 to 75 years with hypertension were recruited from the Banshgram Union of Narial district as part of a cluster-randomized control trial. Current blood pressure was measured as the outcome variable. Associated variables included physical activity at work, travel to and from places, recreational activity, metabolic equivalent task (MET)-min, sedentary time, and awareness of and attitudes towards physical activity. Rasch analysis was used to compute a combined score from the five awareness of and attitudes towards physical activity items and categorized into 0–40 (towards negative attitude), 41–60 score (mixed attitude) and 61–100 (positive attitude). We used a generalised linear model to analyze the data.

**Results:**

Participants (n = 68, 22.1%) who engaged in vigorous-intensity physical activity that causes large increases in breathing or heart rate like carrying or lifting heavy loads, digging or construction work for at least 10 minutes continuously had lower systolic blood pressure (SBP) (mean (95% confidence interval (CI)), 143.6 (140.1, 147.2)) compared to those who did not take part in a vigorous-intensity physical activity (mean (95% CI), 150 (147.6, 152.3)). MET-min less than 600 min/week was significantly associated with higher SBP 153.8 (148.1, 159.6) than MET-min 600–2999 min/week 148.0 (143.0, 152.9) and MET-min>3000 min/week 146.9 (144.5, 149.3), p = 0.001 for trend. Sitting time more than four hours a day was associated with higher DBP 91.4 (89.7, 93.0) compared to those who had sitting time less than fours a day 88.6 (87.1, 90.1). People with positive attitudes were associated with a reduced SBP of 10.6 (0.36, 20.8) mmHg and DBP 5.88 (0.47, 11.3) compared to the people who had a negative attitude towards taking part in physical activity.

**Conclusions:**

**P**articipating in high physical activity and positive attitudes towards physical activity were associated with lower blood pressure levels. Physical activity awareness programs should be implemented to increase awareness of health benefits and increase participation in high physical activity.

## Introduction

Hypertension is a leading risk factor for cardiovascular disease (CVD) and stroke and accounted for approximately 19% of all deaths in 2019 and 9.3% of disability-adjusted life years lost globally [1]. An estimated 1.13 billion people have hypertension, with a significant increase in low- middle-income countries [2]. However, fewer than 10% of people with known hypertension control their blood pressure at a targeted level [3]. Physical inactivity and sedentary behaviour are among the major modifiable risk factors associated with chronic conditions, CVD and all-cause mortality [4–8]. Globally, physical inactivity-related deaths caused by ischemic heart disease (IHD) were 9.9% of 55.14 million deaths in 2017, higher among the aging population and women [9]. Its effects on modifying sympathetic nervous activity and vascular structure and function exercise are significantly associated with improvement in high blood pressure, diabetes, ischemic heart disease and ischemic stroke events [8,10–17]. Indeed, exercise alone has been reported to be associated with a mean blood pressure reduction of SBP/DBP 12/5 mmHg [18], 6±12/3±7 mmHg [19], and 5 to 8 mmHg among adults with hypertension [20]. Previous studies have reported that participation in vigorous-intensity physical activity was associated with a reduced risk of CVD and mortality compared with those undertaking moderate-intensity physical activity [21–23].

Importantly, as life expectancy increases globally, there is a parallel increase in the prevalence of lifestyle-related illness increases, with increments being particularly prominent in low-middle income countries. Indeed, life expectancy has increased by more than 20 years in the last two decades in Bangladesh [24,25], and, coupled with its transitioning from a low- to middle-income country with an increase in urban settlements and economic development [26,27], there is an expectation of increases in the prevalence of non-communicable diseases, including hypertension [28–30]. A systematic review and meta-analyses based on 305,432 participants from 53 studies in Bangladesh reported that 41% of people had hypertension [31]. A cross-sectional study among adults aged ≥30 years in Bangladesh found that 40% of adults had hypertension [32]. Jafar et al. [33] conducted a study among individuals aged 40 years or older with hypertension from Bangladesh, Pakistan and Sri Lanka and reported that 53% had uncontrolled blood pressure among Bangladeshi individuals. Data related to the current study previously reported that two-third of the participants had uncontrolled blood pressure [34].

To increase physical activity and reduce sedentary time for the prevention and control of hypertension [35,36], it is essential to develop population-specific public health intervention programs to raise awareness in maintaining recommended healthy lifestyles [6,35–39]. To the best of our knowledge, no studies in Bangladesh have reported the association of health benefits of physical activity, sedentary lifestyle and attitudes to organize, or participate in physical activity programs with any chronic conditions. A study in India reported that 86% of the participants were physically active, and 92% felt that there were health benefits of being physically active. However, only 25% reported knowledge of any health benefit related to chronic diseases [40]. Although a high proportion of people are physically active, especially in low- middle-income countries [41–43], and have positive attitudes towards participating in physical activity, most people cannot control blood pressure [33,44]. It is also unclear whether being physically active or being aware of the health benefits of physical activity is associated with controlled blood pressure among rural people who have known hypertension.

This study aimed to report: (i) the association of physical activity, and sitting time with blood pressure, and (ii) the association of awareness of and attitudes to physical activity with blood pressure.

## Materials and methods

### Study subjects and sample size

Data from this study are based on 307 participants aged 30–75 years recruited for a cluster randomized control trial from December 2020 to January 2021. The study location was the Banshgram Union which consists of 18 village of the Narail District. Participants from the cross-sectional Bangladesh Population-based Diabetes and Eye Study. Details of this study have been presented in full previously [45,46]. To understand the study location, Bangladesh is a country of over 163 million people divided into 64 districts. Each district is divided into Upazilas (sub-districts), and each Upazila is divided into Unions which consist of 15–20 villages [47]. Eligible participants were adults with clinic-measured blood pressure greater or equal to 130/80 mm Hg and were not on medication or were participants who had controlled blood pressure defined as < 130/80 and were taking antihypertensive medicines for a minimum of six weeks [48]. After communicating with potential participants and receiving consent for their participation in the study, trained data collectors supervised by the local investigator measured the blood pressure to check the eligibility criteria. Upon eligibility, after measuring it, we conducted face-to-face interviews and recorded their blood pressure. Recruitment of eligible participants, measuring blood pressure and conducting face-to-face interviews were performed in nearby community centres or school premises. People who had advanced cardiovascular disease, severe health problems, or did not agree to give written consent were excluded from the study. The cluster RCT was registered (ClinicalTrials.gov, NCT04505150. Registered on 7 August 2020). The authors confirm that all ongoing and related trials for this intervention are registered.

### Sample size

A total of 307 participants (154 in the control group and 153 in the intervention group) was sufficient to provide 95% power at a significance level of 0.01 to detect a minimum difference of 3mm/Hg in blood pressure, assuming a standard deviation of 6 mm/Hg. The sample size for the current research was based on previous research that showed a decrease in 5 mm/Hg systolic blood pressure was associated with a clinically significant reduction in the relative risk of stroke and coronary heart disease events [49].

### Recruitment and data collection

The Organization for Rural Community Development, a local non-government in the Narail district of Bangladesh, facilitated recruitment. The Rural Community Development investigators and trained data collectors communicated with the potential participants over the telephone or by direct contact. Upon establishment of contact with potential participants, they were assessed for inclusion and exclusion and collected their written consent for participation. The Organisation for Rural Community Development investigators and four trained data collectors collected data from face-to-face interviews. An equal proportion of men and women subjects were recruited.

### Primary outcome

The primary outcome measures were systolic and diastolic blood pressure, measured using a calibrated Omron Premium Blood Pressure Monitor Device, Omron HEM-7322. which is reported to produce digital and accurate reading utilising the dual check calibration system. We measured blood pressure twice from the right arm with the person sitting upright. We took the second measure after at least 5 minutes of rest from the first measure and used the average of the two measures. A third measure was taken if the difference between the first two measures was more than 20%, otherwise we took the average of the closest two readings To check the accuracy of the readings, we measured blood pressure from 10 adults using both Omron HEM-7322 and a standard analogue device to compare. Our data showed that the Omron HEM-7322 over-estimated SBP on an average by only 6% (standard deviation (SD) 8%) and DBP by 3% (SD 5%). Given the variation was less than 10% between two devices, we used our data measured by Omron HEM-7322 without further adjustment for overestimate.

### Exposure and confounding variables

The exposure variables were physical activity, sedentary behaviour, and the knowledge of and attitudes towards physical activity.

### Physical activity and sedentary behaviours

We used the Global Physical Activity Questionnaire version 2 (GPAQ-2) [50], developed by the World Health Organisation (WHO) for physical activity surveillance in developing countries, to measure physical activity and sedentary behaviour. The GPAQ-2 comprises 17 questions across three physical activity domains and one sedentary behaviour domain, which include physical activity at work (6 questions), transportation (3 questions), recreational activities (6 questions), and sedentary behaviour (2 questions).

Total physical activity was computed from the three physical activity domains and converted to metabolic equivalent tasks (MET) in minutes (MET-min) per week [51,52]. The MET-min was categorized as insufficient physical activity recommended by WHO with a cut-off of 0 to 600 MET-min, moderate physical activity with a cut-off of 601–3000 MET-min per week and high physical activity with a cut-off of greater than 3000 MET-min per week. Sedentary time was measured as sitting time per day. For this paper we also considered adults at sedentary if they less than 2.5 hours of moderate-intensity exercise, sports or strenuous work per week.

### Awareness of health benefits and attitudes in taking part in physical activity

People who responded that they spent less than 2.5 hours per week were asked the physical activity knowledge, attitudes, and practice questions. Five items were used to assess awareness of the health benefits of physical activity and attitudes towards taking part in physical activity programs. Items were adapted from a smoking cessation motivation questionnaire (Q-MAT) [53]. Awareness was assessed by asking participants whether they were aware of the health benefits of physical activity with possible answers “not at all”, “a little”, “a lot”, and “enormous”. The attitude was assessed by asking the following questions to the participants:

- whether they were interested in taking part in any regular physical activity programs with answers “yes” or “no”,
- at the moment, whether they were considering organising a regular exercise program with their peers with possible answers “yes” or “no”,
- whether they were considering participating in a regular exercise program if it was organized with possible answers “yes” or “no”, and (iv) whether they felt unhappy if they had not done any exercise with the possible answers “never”, “sometimes”, or “often”.

### Confounding factors: Sociodemographic, dietary, and behavioural factors

The confounding variables were sociodemographic factors including age, categorised into less than 40 years who were considered to be young adults, 40 to 59 years who were considered adults and 60 years or older who were considered older adults, gender, the highest level of education–categorized as no schooling, primary to high school (grade 1 to 9), secondary school certificate or any higher-level education. Socioeconomic status- classified as poor, and middle class or rich, assessed according to Cheng et al. [54]. Since most participants had no taxable income, a crude measure of SES was used following Cheng et al. [54] where we asked whether "over the last twelve months, in terms of household food consumption, how would you classify your socio-economic status?" The possible answers were: (i) Insufficient funds for the whole year, (ii) Insufficient funds some of the time, (iii) Neither deficit nor surplus (balance) and (iv) Sufficient funds most of the time. We re-categorised these four categories as poor who had insufficient funds at least some of the time-categories 1 and 2, and middle class or rich who had neither deficit nor surplus (balance) or better SES- categories 3 and 4. Occupations were categorized as: farmer, homemakers, self-managed business, labourers that include digging soils, pulling rickshaw or any laborious works, and employees including government and non-government employees. Dietary habits were assessed by self-reported answers on whether the participants consumed raw salt and fruits and vegetables. The behavioural factor was whether they were current smokers or not.

### Questionnaire preparation

The questionnaire was translated into Bengali separately by a local senior educator and the principal investigator. The two translated versions were then combined and finalised with the questionnaire with an agreement of two translators to use for data collection. The questionnaire was also pilot-tested among ten individuals with hypertension who were not part of this study to assess its comprehension, wording, and appropriateness; no further adjustment was needed. The instrument was used previously to assess awareness and motivation to participate in a smoking cessation program in low- middle-income countries [55].

### Ethics approval and consent to participate

We received the approval for this study by the Swinburne University of Technology Human Research Ethics Committee (Review reference: 20202723–5020). In data collection and management, we followed **relevant guidelines and regulations of the Institution.** The investigators provided written information about the project to the participants. The participants were given the option to discuss the project with local investigators if they had wanted to discuss. The local investigators verbally discussed the project for those who were unable to read or illiterate before collecting their informed consent. All participants were above 18 years of age. Participants were informed that they had full rights to withdraw from the study at any stage if they wished. They were also informed that their decision to participate or not would not influence their relationship with ORCD. **Written informed consent was obtained from all subjects.**

### Statistical analysis

Systolic and diastolic blood pressure were presented by factors related to participants’ characteristics, including sex, age, level of education, occupation, raw salt consumption and smoking status using one-way ANOVA. We presented blood pressure data as mean, and 95% confidence interval (CI) by domain-specific physical activity levels, total physical activity levels measured by MET-min categorized as insufficient physical activity and moderate and high physical activity. Blood pressure also presented by sedentary behaviours. We performed the analyses using a generalised linear model (GLM) after adjustment for age, sex, level of education, socioeconomic status, occupation, smoking status and raw salt consumption status. Blood pressured was also presented by awareness of and attitudes towards physical activity using GLM and adjusting for the same covariates. Due to the small sample, we checked the distribution of data. Associations for the individual item was based on binary categories, recategorized from multiple categorical responses. For example, the item “Do you know physical activity is good for health?” had four categories: “not at all”, “a little”, “a lot”, and “enormous” which was recategorized into two categories such as “not at all or a little” and “a lot or enormous”. Rasch analysis was used to convert categorical responses of the awareness of health benefits and attitudes towards physical activity to an interval scale to create a combined score from all the items. The combined score ranged from zero to 100, categorized into tertile with scores 0–40, defined as negative attitudes towards participating or organizing physical activity, 41–60 defined as neutral attitude and 61–100 as the positive attitude. With reference to positive attitudes, the estimated differences in mean with 95% CI systolic and diastolic blood pressure were obtained using GLM. Statistical software SPSS (SPSS Inc, version 27) was used for the analysis. For Rasch analysis, RUMM (RUMM2030) [56] software was used.

## Results

Table 1 present blood pressure data by sociodemographic, lifestyle and other characteristics of the participants. People aged 60 years or older was associated with higher systolic blood pressure. Other than older age, factors including smoking status, raw salt consumption status, education levels, occupation or hypertensive medication use were not associated with systolic or diastolic blood pressure.

**Table 1:** Blood pressure by sociodemographic, behavioural and other characteristics.

		Number (%)	SBP			DBP		
			Mean	SD	P	Mean	SD	P
Age, years	30–40	46 (15.0)	141	12	0.001	90	8	0.75
	40–59	160 (52.1)	148	18		90	9	
	60 or older	101 (32.9)	153	21		89	11	
Sex	Female	154 (50.2)	150	17	0.24	90	9	0.34
	Male	153 (49.8)	148	20		89	10	
Education levels	No education	99 (32.2)	152	17	0.07	90	9	0.95
	Primary to high school	148 (48.2)	147	19		90	10	
	SSC or above	59 (19.2)	147	18		90	9	
Socio-economic status	Poor	92 (30.0)	150	20	0.41	90	10	0.58
	Middle class	214 (69.7)	148	18		90	10	
Occupation	Farmer	72 (23.5)	146	17	0.34	88	9	0.25
	Homemakers	146 (47.6)	150	17		90	9	
	Employees	53 (17.3)	150	21		89	10	
	Businesspersons	24 (7.8)	149	20		92	12	
Medication use	No medication	163 (53.1)	147	16	0.10	90	9	0.67
	Medication	144 (46.9)	151	21		90	10	
Diabetes	No diabetes	217 (70.7)	149	19	0.42	90	10	0.72
	Diabetes	41 (13.4)	151	18		89	10	
	Unknown	49 (16.0)	146	14		89	9	
Tobacco smoking	Non-smoker	245 (79.8)	149	18	0.34	90	10	0.66
	Current smoker	62 (20.2)	147	20		89	10	
Raw salt consumption status	No raw Salt consumption	66 (21.5)	149	17	0.94	89	8	0.62
	Regular salt consumption	241 (78.5)	149	19		90	10	
Fruit consumption	Maximum 1–2 times per week	46 (15.0)	147	15	0.72	89	7	0.86
	Irregularly	254 (82.7)	149	19		90	10	
	Almost no fruit consumption	7 (2.3)	149	21		92	12	

Table 2 present the systolic and diastolic blood pressure by physical activity at work, commuter, recreation, active hours per week and sedentary behaviour. After adjustment for age, sex, level of education, occupation, smoking status and dietary habits, people who took part in a vigorous activity at work were associated with a lower mean (95% confidence interval (CI)) SBP 145.3 (140.9, 149.8) compared to those who did not take part in vigorous-intensity activity at work mean (95% CI), 150 (147.6, 152.3). Insufficient physical activity according to WHO cut-off values of 0 to 600 MET-min compared to a high level of physical activity with a cut-off of greater than 3000 MET-min per week was significantly associated with higher SBP level means (95% CI) 156.8 (150.2, 163.5) vs. 146.9 (144.5, 149.3), and DBP means (95% CI), 92.5 (89.1, 95.6) vs. 89.1 (87.5, 90.8), respectively. People who travelled to and from the workplace for more than 10 minutes, people who took part in moderate-intensity sports, or spent less than four hours sitting per day were associated with a lower level of SBP before adjustment for covariates.

**Table 2:** Blood pressure by practice in taking part in physical activity at work, travel, recreation, and by sedentary lifestyle.

		Systolic blood pressure		Diastolic blood pressure	
Physical activity	N (%)	Mean (95% CI)*	Mean (95% CI)**	Mean (95% CI)*	Mean (95% CI)**
Total	307 (100)	148.9 (146.8, 150.9)	147.6 (145.1, 150.1)	89.9 (88.8, 90.9)	89.2 (87.9, 90.5)
Activity at work: Vigorous					
No	239 (77.9)	150.3 (147.9, 152.8)	150 (147.6, 152.3)	90.3 (89.1, 91.6)	90.4 (89.1, 91.6)
Yes	68 (22.1)	143.6 (140.1, 147.2)	145.3 (140.9, 149.8)	88.2 (86.0, 90.5)	88 (85.6, 90.4)
P		0.008	0.05	0.11	0.09
Activity at work: moderate intensity					
No	116 (37.8)	152 (148.1, 155.9)	150.8 (147.4, 154.2)	91 (89, 93.1)	91.3 (89.4, 93.1)
Yes	191 (62.2)	147 (144.6, 149.3)	147.8 (145.2, 150.4)	89.1 (87.9, 90.4)	89 (87.6, 90.4)
P		0.02	0.18	0.10	0.06
Travel to and from work, ≥10 minutes					
No	70 (23.0)	153.1 (148.0, 158.3)	151.7 (147.3, 156.2)	91.2 (88.6, 93.9)	91.3 (88.9, 93.7)
Yes	237 (77.0)	147.6 (145.4, 149.8)	148.1 (145.8, 150.4)	89.4 (88.3, 90.6)	89.4 (88.2, 90.7)
P		0.03	0.17	0.18	0.19
Recreation (sports): Moderate intensity					
No	284 (92.5)	149.4 (147.3, 151.6)	149.2 (147.1, 151.3)	89.8 (88.7, 90.9)	89.8 (88.6, 90.9)
Yes	23 (7.5)	141.7 (133.4, 150.0)	145.3 (137.7, 153)	90.7 (86.2, 95.2)	90.8 (86.7, 95)
P		0.05	0.34	0.67	0.64
Sedentary lifestyle: Sitting ≥4 hours/day					
No	164 (53.0)	146.5 (144, 149)	147.5 (144.6, 150.3)	88.7 (87.4, 90.1)	88.6 (87.1, 90.1)
Yes	143 (47.0)	151.6 (148.2, 154.9)	150.6 (147.6, 153.7)	91.1 (89.4, 92.9)	91.4 (89.7, 93.0)
P		0.02	0.14	0.03	0.01
Walk at least 2.5 hours per week					
No	135	152 (148.5, 155.5)	150.8 (147.6, 153.9)	91.1 (89.2, 92.9)	91.4 (89.7, 93.1)
Yes	172	146.4 (143.9, 148.9)	147.5 (144.7, 150.3)	88.9 (87.6, 90.2)	88.7 (87.2, 90.2)
P		0.008	0.13	0.05	0.02
MET-min†					
<600 MET-min	50 (16.2)	156.8 (150.2, 163.5)^a^	153.8 (148.1, 159.6)^a^	93.4 (90.3, 96.6)^a^	92.5 (89.1, 95.6) ^a^
600–2999 MET-min	58 (18.9)	148.9 (144.5, 153.3)^b^	148.0 (143.0, 152.9)^b^	90.4 (87.8, 93.1)^b^	90.9 (88.2, 93.6) ^b^
≥3000 MET-min	199 (64.8)	146.9 (144.5, 149.3)^b^	146.9 (143.9, 150.0)^b^	89.0 (87.8, 90.3)^b^	89.1 (87.5, 90.8) ^b^
P for trend		0.001	0.05	0.02	0.03

*Estimated mean with 95% confidence interval (CI) (unadjusted model)

** estimated mean with 95% CI adjusted for age, sex, level of education, occupation and diabetes status.

† subscript with different letters, e.g., “a” and “b” indicate a significant difference at least at a 5% level of significance, the same letter “b” indicates non-significant difference.

Table 3 presents the blood pressure by awareness of and attitudes towards taking part in physical activity. After adjustment for covariates, people who were aware of the health benefits for physical activity were more likely to have a lower level of both SBP mean (95% CI) 145.7 (138.5, 152.9) vs 151.9 (143.8 160.1), p = 0.02 and DBP (mean (95% CI) 89.8 (85.9, 93.7) vs 94.2 (89.8, 98.7), p = 0.03 compared to those who were unaware of health benefits of physical activity. However, attitudes to organise regular exercise programs or participating in any regular physical activity or unhappiness for not participating in any regular exercise programs were not associated with blood pressure.

**Table 3:** Blood pressure by knowledge of and attitudes towards taking part in physical activity among people who were physically less active.

		Systolic blood pressure	Diastolic blood pressure
Knowledge of and attitudes towards physical activity items	No at risk	Mean (95% CI)*	Mean (95% CI)*
Total	135	152 (148.5, 155.5)	91.1 (89.2, 92.9)
†Do you know physical activity is good for health?			
Not at all or a little	71	151.9 (143.8 160.1)	94.2 (89.8, 98.7)
A lot or enormous	64	145.7 (138.5, 152.9)	89.8 (85.9, 93.7)
P		0.02	0.03
Are you interested to take part in any physical activities for 2.5 hours per week			
No	20	149.7 (139.3, 160.1)	92.2 (87.3, 97.1)
Yes	115	152.4 (148.7, 156.1)	90.9 (88.9, 92.9)
P		0.59	0.64
At the moment, do you consider to organise regular exercise program with your peers			
No	104	152.8 (148.6, 156.9)	91.7 (89.6, 93.8)
Yes	31	149.5 (142.9, 156.1)	89 (85.1, 92.9)
P		0.44	0.23
At the moment, do you consider participating in regular exercise program if it is organized			
No	101	154.3 (150.3, 158.2)	91.6 (89.4, 93.7)
Yes	34	145.3 (138.1, 152.5)	89.7 (86, 93.4)
P		0.02	0.38
Do you ever feel unhappy if you do not do any exercise?			
Never	42	151.8 (146.2, 157.5)	92.4 (88.9, 95.9)
Sometimes or often	93	152.1 (147.6, 156.5)	90.5 (88.3, 92.7)
P		0.77	0.29

*Estimated mean with 95% confidence interval (CI) (unadjusted model).

**†** estimated mean with 95% confidence interval (CI) adjusted for age, gender, education levels and occupation.

Table 4 presents the blood pressure by the combined score of the awareness of and attitudes towards physical activity. People with positive attitudes defined with the top 33% of the converted score was associated with a significant lower SBP (mean difference (95% CI) 10.6 (0.36, 20.8) and DBP (mean difference (95% CI) 5.88 (0.47, 11.3)) from the people who had negative attitudes defined with the lowest 33% of the converted score before adjustment for covariates. After adjustment for age, sex, level of education, occupation, smoking status and dietary habits association of positive attitudes with blood pressure attenuated.

**Table 4:** Blood pressure by converted and combined knowledge of and attitudes towards taking part in physical activity score in 135 people who were less physically active less than 2.5 hours/week.

Converted		Systolic Blood Pressure, mmHg, ΔMean (95% CI)*	
Attitudes scores (combined)	N (%)	Model 1*	Model 2**
Tertile 1: 0–40	33	10.6 (0.36, 20.8)	7.37 (-4.11, 18.9)
Tertile 2: 41–60	73	4.80 (-3.64, 13.2)	4.55 (-4.12, 13.2)
Tertile 3: 61–100 (Ref.)	29	0	0
		Diastolic Blood Pressure, mmHg, ΔMean (95% CI)	
Combined score	No at risk	Model 1	Model 2
Tertile 1: 0–40	33	5.88 (0.47, 11.3)	5.31 (-.93, 11.56)
Tertile 2: 41–60	73	2.16 (-2.30, 6.61)	2.08 (-2.63, 6.80)
Tertile 3: 61–100 (Ref.)	29	0	0

*Estimated effect size, mean with 95% confidence interval (CI) changes compared to people who had 61 to 100% aware and had positive attitudes towards physical exercise. *Model 1: Unadjusted model, and **Model 2: Age, sex, education and occupation adjusted.

## Discussion

With increased prevalence of hypertension in low- middle-income countries, physical activity has a significant role, either as a single factor or additive factor in combination with antihypertensive medication and other established risk factors in the treatment of hypertension. Key important findings from this study were that moderate to high physical activity levels were associated with a lower blood pressure level. Awareness of the health benefits of physical activity was associated with a lower level of blood pressure, and people with positive attitudes towards physical activity had 5 to 10 mmHg lower blood pressure levels compared to those who had negative attitudes toward taking part in physical activities.

Association of physical activity (measured using the GPAQ questionnaire [50]) with lower blood pressure levels is consistent with previous studies [21,23,57]. The current study with cross-sectional data found that participating at the WHO minimum recommended levels of physical activity level was associated with lower blood pressure; at least seven mmHg SBP and three mmHg DBP. The magnitude of effect is consistent with previous studies [10,11,14]. Börjesson et al. [10] conducted a narrative review of 27 RCTs and reported that medium-to-high intensity aerobic activity was associated with reducing mean blood pressure by 11/5 mmHg. In a study of dose-response of physical activity in controlling blood pressure, Ishikawa-Takata [14] reported that 61–90 minutes/week exercise was associated with a significant reduction in SBP compared to 30–60 min/week physical activity. Powel et al. [21] reported that any physical activity was associated with health benefit and 150–300 min/week of moderate-intensity activity were associated with substantial health benefits. International physical activity guidelines recommend that adults should engage in at least 30 minutes a day of at least moderate-intensity activity [58]. Climie et al. [59] had provided more insight into the association of domain-specific physical activity with cardiovascular diseases through a large cross-sectional. The study reported that physical activity related to occupation was associated with worse neural baroreflex sensitivity. In contrast, sports physical activity was associated with better neural baroreflex sensitivity. The findings indicate that different mechanisms of associations could be between domains of physical activity and blood pressure control.

In our study, the association of vigorous-intensity activity with a lower blood pressure level was persistent after covariate adjustment. Previous studies [60–62] reported similar findings. However, an association of moderate-intensity physical activity with lower SBP was attenuated adjustment for covariates, especially after adjustment for level of education and occupation. The vigorous-intensity activity was five times higher among farmers who were five times less likely to be educated than the employees or businesspeople. This can be why adjustment for education and occupation has a negligible effect on the significant association between vigorous-intensity activity and lower blood pressure levels. In contrast, attenuation of significant association of moderate-intensity activity with a lower blood pressure level can be explained that moderate-intensity activity is equally likely among all professions. Also, people with higher education took part mainly in moderate-intensity physical activity. However, according to Climie et al. [59], further investigation is needed to understand better the associations’ mechanisms of domain-specific physical activity levels.

Our study demonstrated that awareness of health benefits and positive attitudes towards participation in physical activity was associated with lower blood pressure levels. Knowledge of the health benefits of physical activity is expected to change people’s attitudes towards physical activity in terms of association with lowering blood pressure [40,63,64]. Veluswamy et al. [40] conducted a cross-sectional study to assess the awareness of chronic disease-related health benefits of physical activity, which reported that people who perceived leading an active lifestyle were more likely to be involved. Therefore, this is essential to develop various population-specific health promotion programs to increase awareness and change in attitudes in maintaining recommended healthy lifestyle that includes participating in regular physical activity to potentially control blood pressure [6,35–39]. Previous studies have shown that lifestyle changes may also depend on awareness and knowledge to initiate change to maximize its benefits [65–67].

Physical inactivity is a key modifiable risk factor for hypertension [68]. Managing hypertension by changing one’s lifestyle could be the best impactful practice to supplement one of the significant barriers in the health system- the shortage of qualified doctors, especially in rural areas [69,70]. The present study highlights the potential for increasing moderate-to-vigorous intensity physical activity for controlling blood pressure; however, in low-middle-income countries access to facilities to participate in such activities is limited. Moreover, those countries may not be aware of health benefits that moderate to vigorous-intensity activity offers for controlling blood pressure [21–23].

Our study has several strengths: firstly, data were collected in person. The gender balance was equal. However, a limitation of this study was that it was conducted in a single district, limiting generalizations at the national level. However, the rural population in terms of socio-demographic and education level is similar in Bangladesh [71]. A further limitation of the study is the unavailability of the body mass index data. Due to cultural and religious barriers in measuring the height and weight of women, especially during the COVID period, the ethics approval was challenging. Thus, BMI data are absent in this study. Another limitation was measuring blood pressure with an automated device. We checked the accuracy of the readings using a standard analogue device from a sub-sample and found the accuracy level was high. Also, the Omron HEM-7322 has reported to provide validated measures of blood pressure [72]. Another limitation was that we had an arbitrary cut-off of intention to physical activity score into three categories which lacks observing the association in a linear scale.

## Conclusions

Moderate to vigorous intensity physical activity level was associated with lower blood pressure levels. People who were aware of the health benefits of physical activity and had positive attitudes to participate in physical activity were associated had lower BP levels. Given the increasing prevalence of hypertension, physical activity has a significant role as a single or additive treatment for hypertension. Various health promotion programs can be organized at a community level to increase motivation and attitudes towards physical activity and act upon it to control blood pressure.

## Supporting information

S1 Data Lowering blood pressure_PLOS One. (SAV)

## Abbreviations

ORCD: for Rural Community Development
SES: Socioeconomic status
CI: Confidence Interval
OR: Odds Ratio
CVD: : cardiovascular disease
GPAQ-2: Global Physical Activity Questionnaire version 2
WHO: World Health Organisation
MET-min: metabolic equivalent tasks (MET)-Minutes
Q-MAT: smoking cessation motivation questionnaire
GLM: generalised linear model
SBP: Systolic blood pressure
DBP: Diastolic blood pressure
